# Automated diagnosis of pancreatic mucinous and serous cystic neoplasms with modality-fusion deep neural network using multi-modality MRIs

**DOI:** 10.3389/fonc.2023.1181270

**Published:** 2023-09-19

**Authors:** Gong Zhang, Weixiang Chen, Zizheng Wang, Fei Wang, Rong Liu, Jianjiang Feng

**Affiliations:** ^1^ Faculty of Hepato-Biliary-Pancreatic Surgery, the First Medical Center of Chinese People’s Liberation Army (PLA) General Hospital, Beijing, China; ^2^ Department of Automation, Tsinghua University, Beijing, China; ^3^ Senior Department of Hepatology, Fifth Medical Center of the PLA General Hospital, Beijing, China

**Keywords:** deep neural networks (DNN), severe cystic neoplasms (SCN), mucinous cystic neoplasms (MCN), automatic computer diagnosis (ACD), computer-aided diagnosis (CAD), modality fusion

## Abstract

**Background:**

Pancreatic cystic neoplasms are increasingly diagnosed with the development of medical imaging technology and people’s self-care awareness. However, two of their sub-types, serous cystic neoplasms (SCN) and mucinous cystic neoplasms (MCN), are often misclassified from each other. Because SCN is primarily benign and MCN has a high rate of malignant transformation. Distinguishing SCN and MCN is challenging and essential.

**Purpose:**

MRIs have many different modalities, complete with SCN and MCN diagnosis information. With the help of an artificial intelligence-based algorithm, we aimed to propose a multi-modal hybrid deep learning network that can efficiently diagnose SCN and MCN using multi-modality MRIs.

**Methods:**

A cross-modal feature fusion structure was innovatively designed, combining features of seven modalities to realize the classification of SCN and MCN. 69 Patients with multi-modalities of MRIs were included, and experiments showed performances of every modality.

**Results:**

The proposed method with the optimized settings outperformed all other techniques and human radiologists with high accuracy of 75.07% and an AUC of 82.77%. Besides, the proposed disentanglement method outperformed other fusion methods, and delayed contrast-enhanced T1-weighted MRIs proved most valuable in diagnosing SCN and MCN.

**Conclusions:**

Through the use of a contemporary artificial intelligence algorithm, physicians can attain high performance in the complex challenge of diagnosing SCN and MCN, surpassing human radiologists to a significant degree.

## Introduction

Pancreatic cystic neoplasms (PCN) are a diverse group of tumors in the pancreas that differ in terms of clinical presentation and prognosis. The four main subtypes are mucinous cystic neoplasms (MCN), serous cystic neoplasms (SCN), intraductal papillary mucinous neoplasms (IPMN), and solid pseudopapillary neoplasms (SPN). Although PCN is rare, accounting for only 1-2% of pancreatic tumors, advances in medical imaging and increased awareness of population health screening have led to a rise in their detection rate (1, 2).

Management of different types of PCN varies based on specific principles and as outlined in clinical guidelines. Surgery is typically not necessary for asymptomatic SCN. However, resection of SCN is recommended when patient-related factors, cyst-related factors, and clinical guidelines indicate a potential for complications or significant symptomatic manifestations. Common indications for SCN resection include: 1) large cyst size (greater than 4 cm) which increases the risk of complications such as rupture or malignant transformation; 2) presence of symptoms such as abdominal pain, compression of adjacent structures, or gastrointestinal symptoms; 3) suspicion of malignancy, as rare cases of malignant transformation have been reported in SCN; 4) uncertainty in the diagnosis of SCN or inability to definitively rule out other types of pancreatic cysts, where resection provides a definitive diagnosis and avoids potential complications. It is important to consider individual patient characteristics, expert clinical judgment, and engage in discussion with the patient and their healthcare provider when making decisions regarding SCN management, including the choice for resection.

On the other hand, other cystic pancreatic tumors require close monitoring or surgical resection due to their potential for malignancy (3–5). Given the challenges and high risks involved in pancreatic surgery, it is crucial to minimize unnecessary surgeries and avoid delays caused by prolonged observation periods. Therefore, accurate differentiation of various PCN through preoperative imaging becomes imperative. Among the four types of PCN, SCN and MCN hold the highest importance in facilitating differential diagnoses. Although SCN and MCN have characteristic presentations, the differential diagnosis becomes challenging when dealing with atypical morphology or small size, as the imaging appearances are more similar. According to the literature, the classification accuracy in such cases can be as low as 47%-58.6% (6, 7).

Many radiologists are working to find ways to diagnose them better. Previous studies have shown that many essential features associated with cystic tumors can be observed on CT and used as a basis for diagnosis. The number, size, location, calcification, tissue enhancement of the neoplasms’ capsule, and the presence and thickness of the capsule wall can help diagnose PCN (8). However, the diagnostic performance of these morphological features is not stable and depends on the judgment of radiologists.

In recent years, the so-called radiomics method has proposed and applied more objective image-based features in diagnosing breast diseases (9). Then, the researchers used similar methods to analyze PCN. Wei et al. (10)proposed a radiomics-based plan to screen SCN using multidetector row computed tomography (MDCT) images and yield a performance of AUC=0.767 in the data set of 200 patients. Yang et al. (11) achieved the performance of AUC 0.777 in SCN and MCN differentiation using the radiomics method and achieved the result of AUC 0.893 after adding the morphological features. However, radiomics is still a half-automated method that needs doctors to circle the area of cysts first. Besides, rare publications try to use Magnetic Resonance Imaging (MRI) to diagnose SCN and MCN. Unlike CT or MDCT, MRI contains more helpful information, which may help analyze better.

Deep learning methods usually work better than traditional machine learnings, extracting the best features and optimizing classification weights according to objective functions. Many previous studies have made breakthroughs in pancreatic anatomy and diseases, but rare publications have focused on SCN and MCN. Zhou et al. (12, 13) realized precise pancreas segmentation through the deep network and accurate cysts segmentation. Hussein et al. (14) achieved the auxiliary diagnosis of IPMN with MRI. Lalonde et al. (15) further proposed the automatic diagnosis of IPMN based on two MRI-weighted deep learning schemes. For diagnosing SCN and MCN, Chen et al. (16) proposed that PCN-net realized automatic diagnosis based on T1 and T2 modalities. Some former works tried using multi-modality to diagnose, but the number of included modalities was only T1 and T2. For a study of SCN and MCN MRI, the researchers took a series of different modalities. The value of using more modalities in diagnosis is unknown.

To fill the research gap, we proposed an automated method with deep networks to diagnose SCN and MCN. Seven modalities of MRI were included in our experiments to compare their performances. Besides, different modality fusion methods were tested, and the optimized fusion method was proposed.

## Materials and methods

### Aims and objectives

**1)** This research presents a novel fully automated diagnostic system for SCN and MCN, boasting high accuracy and offering practical assistance to radiologists.
**2)** Given the varying information yielded by different patterns, our study aims to assess the performance of individual MRI patterns using a deep learning network, enabling quantitative evaluation of their respective contributions to the diagnosis of SCN and MCN.
**3)** Our objective is to investigate the optimal fusion method for integrating diverse MRI modalities with the ultimate goal of establishing the most effective model for diagnosing SCN and MCN.

### Data source and study population

This study collected data from pancreatic SCN and MCN patients who underwent surgery in a large capacity central hospital, from February 2016 to March 2019. The study focused on lesions primarily located in the pancreatic head and body.

The study’s inclusion criteria were as follows: 1) Patients who had undergone a comprehensive MRI examination utilizing all seven modalities (The moalities of these seven MRIs are described in detail in the next section Multi-modality MRI protocols) 2) The diagnosis of SCN or MCN was confirmed through postoperative pathological examination. Patients were excluded from the radiomic analysis if they met the following criteria: 1) Samples with lost or damaged image data; 2) The pathological diagnosis was mucinous cystadenocarcinoma.

A total of 69 patients were included in the cohort, with 33 cases diagnosed as SCN (including microcystic 19, macrocystic/unilocular 8, micro-macrocystic 4, pseudosolid variants 2) and the remaining 36 cases identified as MCN. The hospital ethical committee approved the study, and written consent was obtained from the patients before the surgery.

### Multi-modality MRI protocols

**Figure 1:**
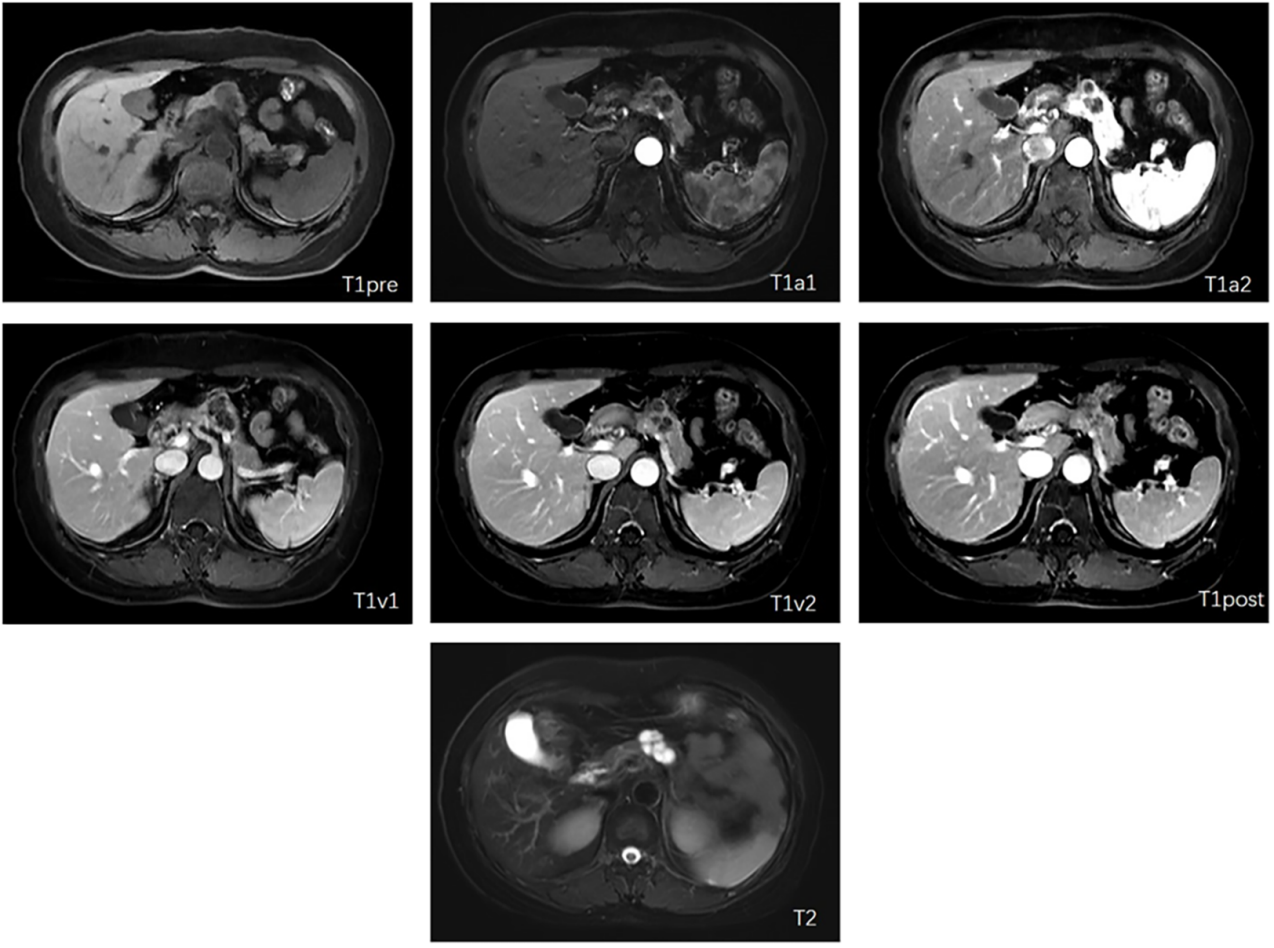
Examples of included seven modalities **(**The letter in each subfigure is notation of modalities for the figure).

Dynamic enhanced MRI scanning with Gd-DPTA was performed for image acquisition, preprocessing, and segmentation on either 3.0T platforms, such as the Discovery MR750 (GE, USA), Magnetom Skyra (SIEMENS, Germany), and uMR770 (United imaging, China), or 1.5T platforms including Multiva (Philips, Netherlands) and uMR560/570 (United imaging, China). The image includes the following sequence: 1) Axial fat-saturated T2-weighted imaging (T2WI); 2) Axial diffusion-weighted imaging (DWI) with B=800 m/s^2; 3) Axial T1 opposed-phase imaging; 4) Axial dynamic enhanced T1-weighted imaging (T1WI) at the arterial phase; and 5) Axial dynamic enhanced T1-weighted imaging (T1WI) at the portal venous phase. We finally selected T1 weighted and T2 weighted MRI, and T1 weighted MRI was divided into six different modalities, the plain scan phase, early arterial phase, late arterial phase, early venous phase, late venous phase, and delayed phase, according to the scan timepoint after injected contrast agents. We denoted them as T1pre, T1a1, T1a2, T1v1, T1v2, T1post, and T2. Different modalities show different information which can help diagnose better. All the patients in this study had seven other modalities of MRI, and each modality was 3D volume (**Figure 1** **)**. Because the resolution of diffusion-weighted imaging (DWI) is low and usually used as a complement to contrast-enhanced MRI, DWI is not selected in this study which is at the preliminary exploration stage for the neural network and deep learning methods of SCN and MCN recognition.

Included MRIs were collected by different machines, resulting in different settings in spacing. Spacings of our T1 weighted MRIs ranged from [0.78 0.78 2.59] mm to [1.04 1.04 2.50] mm, while that of T2 weighted ranged from [0.625 0.625 4.80] mm to [0.88 0.88 8.40] mm. T2 weighted images had larger spacing along the Z axis. For better analysis, all images were resized and normalized.

### Outcome measures

The proposed deep network produces the diagnosis probability of each category for a slice. To obtain a patient-level diagnosis, an extra slice-fusion block was proposed. We did three experiments to evaluate diagnosis performances of every single modality, fusion modalities, and patient-level arrangements. For all experiments, sensitivity (accuracy of MCN), specificity (accuracy of SCN), total accuracy, and area under curves of receiver operator (AUC) were calculated.

The diagnosis probabilities had been normalized, so that chances of SCN and MCN sum up to 1. Thresholded by 0.5, predicted results were obtained. We regarded MCN as positive samples, so sensitivity was defined by True-MCN/(True-MCN+False-SCN). Similarly, specificity was determined by True-SCN/(True-SCN+False-MCN). (True-MCN+True-SCN)/All-samples limited accuracy. Sensitivity and specificity are trade-offs, while the receiver operator curve (ROC) can help measure the performances. By changing the thresholds, ROC is obtained by all working points with every entry. The AUC is the area under the curve, and the larger AUC means better diagnosis results. In our 4-fold cross-validation, all metrics mentioned above had been obtained in all folds and averaged.

### MRI data preprocess

**Figure 2:**
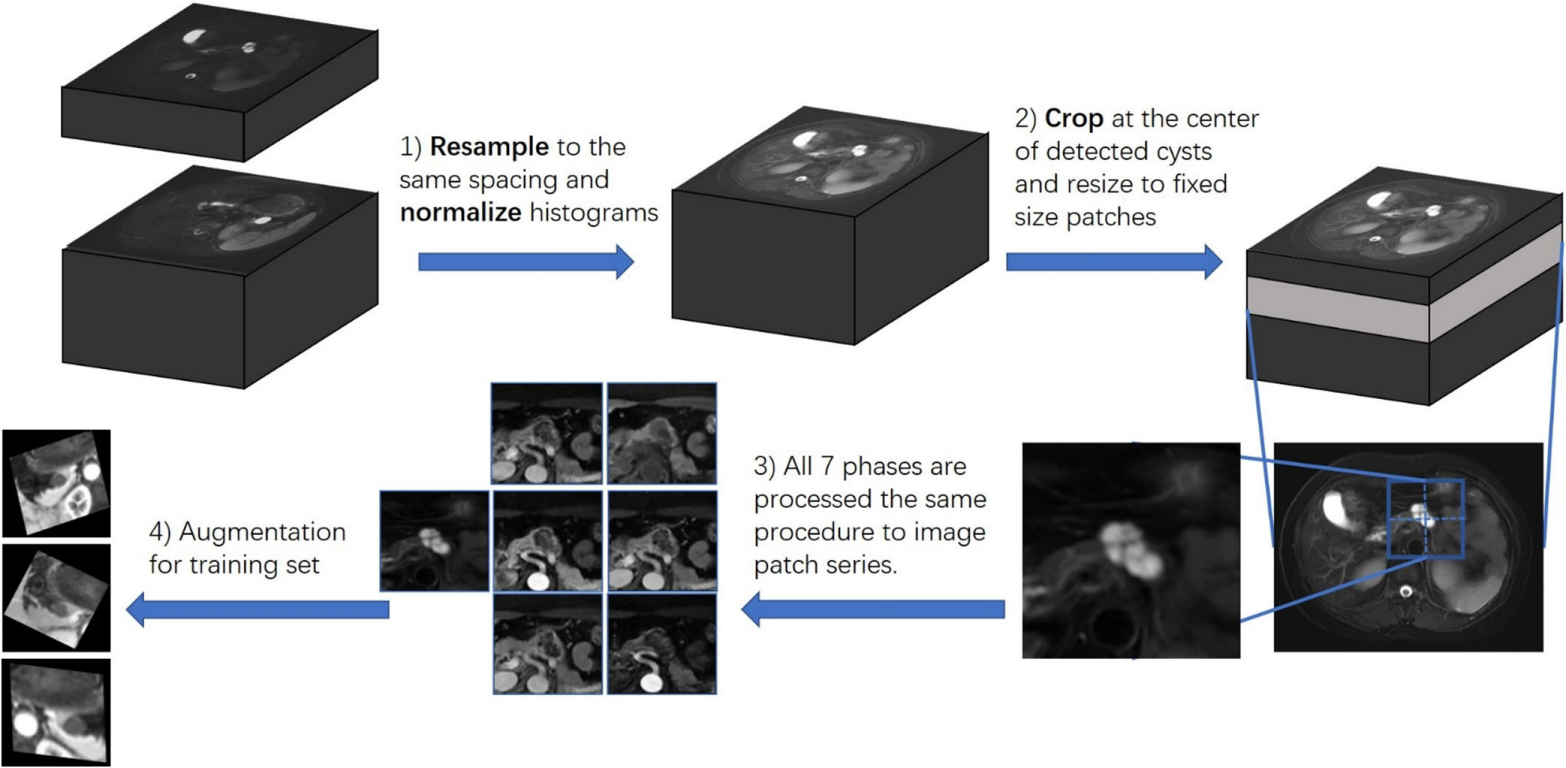
Workflow of data preprocess.

Since the raw data were in different spacing and the gray value ranges from -1700 to 2048, we firstly resampled all MRIs into 1x1x1 mm per voxel spacing and normalized all gray values into [-1,1]. The gray value was truncated with different windows of different modalities. Then, to better analyze the performances of other modalities, we manually located cysts on every modality and cropped them out with a box with a side length of 80 voxels. The cropped images were transformed into slices and paired with pieces at the same position as other modalities. The image pairs were training and test samples of our experiments. For the training set, data augmentations were done. The whole process was shown in **Figure 2**.

### Deep learning networks

**Figure 3:**
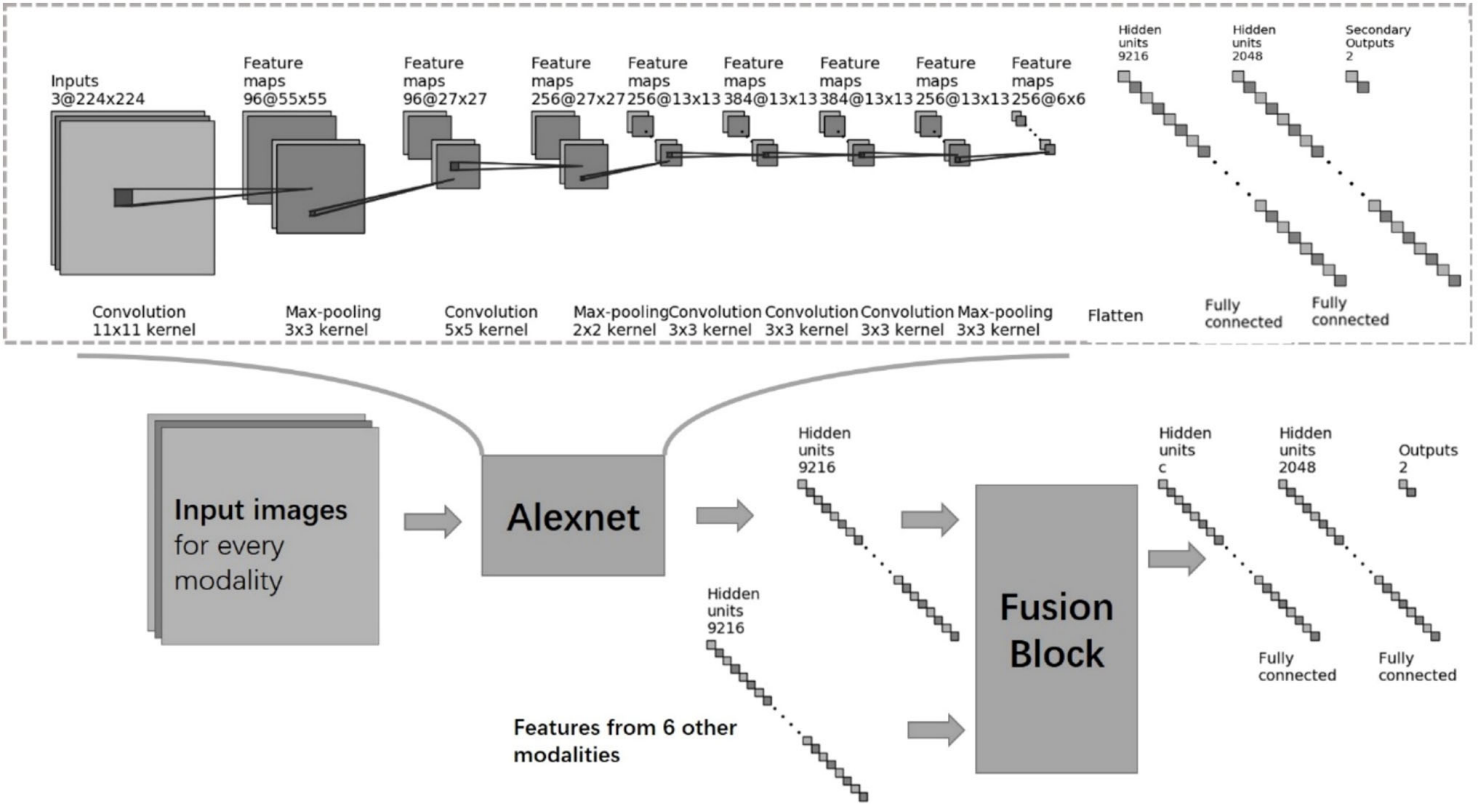
Architecture of our deep networks.

Deep Neural Networks (DNN) have been used in many questions and proved that deep features could work better than hand-craft-designed features. Our DNN can be roughly divided into body parts and head parts. The proposed network used the convolutional layer of Alexnet (17) as the body part and truncated the fully connected layer of it. For the head part, a modality fusion block was proposed, and fully-connected layers were set up as a classifier. The whole network can be trained end to end. Directly inputted the seven modalities image, the classification results can be obtained from the output end, and the entire network is shown in **Figure 3**. We used a network pre-trained on the ImageNet (18) dataset, a vast image set containing 14,197,122 natural photos. Natural images were captured in RGB-channel, which is much different from medical images. However, we still use the parameters pre-trained on ImageNet because parameters pre-trained on medical images are not available. It turned out to be worse if we did not use pre-trained parameters.

**Figure 4:**
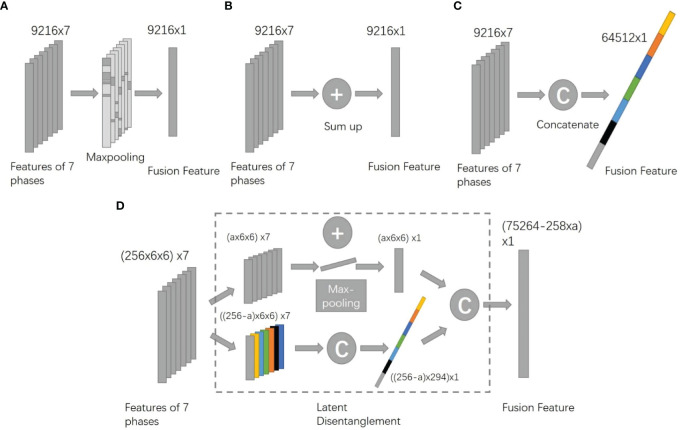
Architectures of different fusion methods **(A)**. MAX method. **(B)** SUM method. **(C)** CAT method. **(D)** the proposed DIS method).

The head part of the deep network proposed contained a fusion block and a classifier composed of two layers of fully connected networks. We have tested different types of fusion blocks, including CAT fusion which concatenated features from seven modalities, SUM fusion which summed up characteristics from seven modalities, and MAX fusion which calculated the max value of each part of seven modalities and kept the maximums as fusion results (**Figures 4A–C**). In addition, a naïve method that diagnoses every modality and does majority voting was also tested.

The proposed fusion block combined the benefit of CAT and SUM fusion. It disentangled features into two spaces, the appearance space where all modalities have different information and the content space that all seven modalities share. For appearance features, the block concatenated them while it summed up content features. (**Figure 4D**). To better train the body part of the network, we added secondary outputs, as shown in **Figure 3**, and also plugged in the same classifier behind each module feature to monitor the characteristics of each model.

In the data set of 1835 groups of 7 modalities images, we conducted four-fold cross-validation, with the test set size of n=458 or n=459 and the training set size of n=1374 or n=1375 for each fold. The division was always done at the patient level, meaning the same patient’s images can only appear in a single fold. The training set for each training session is augmented with data.

Since our model worked on slice-level and when diagnosing in practice, we need patient-level results; we proposed SVM to fusion slices’ results into one final result.

## Results

Sixty-nine patients were included in our cohort. Thirty-three cases were diagnosed as SCN, and the other 36 were MCN. Some other characteristics are presented in **Table 1**. According to the statistical analysis, those characteristics had little correlation with diagnosis results **Table 1**. Due to the small cohort for deep learning, we took 4-fold cross-validation in our experiments. When selecting validation cohort in every fold, the ratio of categories was kept. The male-to-female ratio of SCN was 0.38, MCN was 0.29, and gender was not associated with the diagnosis (p=0.78). The average age of SCN patients was 53.03 years old, and that of MCN patients was 50.16 years old, which was not correlated with the diagnosis result (p=0.33). The median maximum size of cystadenoma in patients with SCN and MCN was 2.80 cm and 3.00 cm, respectively, and there was no statistical correlation with the diagnosis result (p=0.10).

**Table 1:** Characteristics of included subjects.

	SCN group	MCN group	*p* value
	n=33	n=36	
**Age [mean (year) ± SD]**	53.03 ± 9.93	50.16 ± 13.98	0.33
**Gender [n (%)]**			0.78
**Female**	24 (72.7)	28 (77.8)	
**Male**	9 (27.3)	8 (22.2)	
**Tumor size[media (cm), IQR]**	2.80 (1.85,4.38)	3.00 (2.00,5.00)	0.10

Seven different modalities showed different results in the diagnosis. **Table 2** shows the result of using one single modality for diagnosis. When testing a single modality, we directly took the secondary outputs as diagnosis results. Average Result (AR) can be regarded as a simple result-level modality fusion by majority voting seven modalities. This naive fusion method can only slightly outperform the best single modality. The results showed that T1post has the highest performance with an AUC of 0.84. The AUC was even better than the naïve fusion method, AR, which indicated that the fusion method should be carefully designed; otherwise, the performance might not promote. The proposed method yielded 75% accuracy, which was 4% higher than human radiologists.

**Table 2:** Results of every single modality.

Modality	Specificity	Sensitivity	AUC
**T2**	0.64 ± 0.08	0.77 ± 0.09	0.78 ± 0.08
**T1pre**	0.50 ± 0.09	0.71 ± 0.18	0.66 ± 0.12
**T1a1**	0.72 ± 0.14	0.74 ± 0.17	0.78 ± 0.11
**T1a2**	0.74 ± 0.23	0.77 ± 0.12	0.81 ± 0.09
**T1v1**	0.74 ± 0.23	0.74 ± 0.15	0.80 ± 0.05
**T1v2**	0.71 ± 0.21	0.74 ± 0.15	0.79 ± 0.07
**T1post**	**0.81 ± 0.16**	0.74 ± 0.13	**0.84 ± 0.06**
**Average Results**	0.75 ± 0.26	**0.78 ± 0.14**	0.80 ± 0.08

The results were computed by averaging 4-fold results, and the ranges of all results were also shown. Bold means most effective and plays an emphasis role.

**Table 3** shows the results of different fusion methods. Except for MAX, SUM, and CAT fusion, we also tested feature-level and image-level fusion effects (IMG-F). Image-level fusion fuses other modalities before processing through the networks by concatenating all images in a series into a 7-channel image. However, merging results is much too naive for the results are separately computed, and combining images is much too shallow for image pixels without much information to be concatenated together. The proposed method, DIS, has a critical hyper-parameter, the length of appearance feature. We have tested many different settings and showed the 128 and 200 ones, which were around the optimized point in our experiments. The results showed that the disentanglement method with 128 appearance features had the best performance with an AUC of 0.85.

**Table 3:** Results of different modality fusion methods.

Fusion method	Specificity	Sensitivity	AUC
**IMG-F**	0.70 ± 0.18	0.66 ± 0.13	0.75 ± 0.12
**SUM**	0.75 ± 0.21	0.80 ± 0.15	0.83 ± 0.09
**MAX**	0.75 ± 0.17	0.82 ± 0.13	0.83 ± 0.11
**CAT**	0.77 ± 0.22	0.76 ± 0.18	0.82 ± 0.09
**Dis-128**	**0.82 ± 0.20**	**0.84 ± 0.12**	**0.85 ± 0.11**
**Dis-200**	0.78 ± 0.21	0.82 ± 0.13	0.84 ± 0.11

The results were computed by averaging 4-fold results, and the ranges of every results were also shown. Bold means most effective and plays an emphasis role.

**Table 4** shows patient-level results in comparison with human radiologists. The radiologist’s assessment was conducted by two experienced board-certified radiologist with expertise in neuroimaging. They have over 10 years of experience interpreting similar types of images and have undergone specialized training in this field. They were asked to review the slices without help from other information about the patients and make the diagnosis. Experts were asked to view the whole volumes just like in the practice. We also compare the results with the traditional radiomics^8^ method and human experts described in the former section. Radiomics method uses many manual designed features, such as intensity, shape, texture, wavelet, and LOG features, and has been found helpful in several clinical areas, such as oncology and cardiology. The proposed method outperformed all other methods with high accuracy of 75% and an AUC of 0.83.

**Table 4:** Results of patient level diagnoses.

Method	Accuracy	AUC
**Radiologist 1**	0.68	0.73
**Radiologist 2**	0.71	0.76
**Radiomics+SVM**	0.58	0.62
**Proposed+majoraty voting**	0.68	0.80
**Proposed+SVM**	0.75	0.83

The results were calculated from the whole cohort fold -by-fold for algorithms, while radiologists directly read all data from coho.

## Discussion

Pancreatic cystic neoplasms (PCN) are a group of pancreatic tumors with widely varying clinical presentation and prognosis. Four cystic pancreatic neoplasms include MCN, SCN, Intraductal Papillary Mucinous Neoplasm (IPMN), and solid pseudopapillary neoplasms (SPN). Misclassification between SCN and MCN is common. However, the SCNs are mostly benign, and MCNs have a high malignancy rate. Therefore, distinguishing SCNs and MCNs is challenging and essential. Currently, there is an effective tool for distinguishing between SCNs and MCNs, which is the technique of endoscopic ultrasound biopsy. Previous research has shown that endoscopic ultrasound biopsy has a high level of accuracy in histological examination. However, because it is an invasive procedure, it may increase the risk of associated complications (19–21). Therefore, we aim to differentiate SCN from MCN using a non-invasive imaging approach through deep learning technology.

MRI images have different weight settings and scan phases, which have a wealth of information and can reflect the functional features of the lesions in addition to morphological features. However, previous work has often enabled imaging physicians to make more accurate judgments of the diagnosis of PCN using the combined analysis of different MRI images.

In the deep network proposed in this paper, a cross-modal feature fusion structure was innovatively designed. The seven different modal depth features obtained were fused into one combined part to realize the classification of modal fusion. Our proposed method achieved good results in diagnosing SCN and MCN with an accuracy of 75% and AUC of 0.83, which is even higher than human radiologists. Besides, the method works efficiently and automatically. Our experiments also estimated the performances of different MRI modalities quantitively. Confidence intervals of our results were defined as the ranges of results in 4 folds, which were a little giant because the training and validation sets are divided separately. Some folds might be very easy, while some are hard. However, the average results shown in the table can be regarded as stable performances for the whole cohort.

### Different information of different modalites

The typical morphology of SCN is in a multicystic shape and with a lobulated contour. SCN usually has a thin wall and central scar in about 30% of cases. While the MCN are primarily solitary, with thick and uneven cyst walls, there are wall nodules, eggshell-like calcification, and segregation in the cyst. The wall nodules and fibrous septations between the cysts would begin to enhance after the injection of Gadolinium and enhance greatly on delayed contrast-enhanced modality.

This study’s T1post had the best performance, significantly outperforming the other contrast-enhanced modalities. To some degree, this result coincides with human experiences that delayed contrast-enhanced modality is a better and proper phase for PCN diagnosis.

T1pre had the worst performance, which could hardly provide practical information for the diagnosis, and T2 had a similar arrangement with other contrast-enhanced modalities. Indeed, there is no contrast-enhanced effect of Gadolinium in the T1WI plain scan and T2WI phase. However, T2WI images could clearly demonstrate the general appearance of the SCN or MCN owing to the cystic fluid’s high signal intensity and the low signal intensity in the central scar and calcification of SCN.

### Promotion from multi-modality fusion

Modality fusion is an essential topic in computer science and medical image analysis. For medical images, especially MRI, a series of volumes in different settings are captured as a normal process. To distinguish between MCN and SCN, experienced radiologists will look at and compare many other MRI modalities, which encourages us that fusion of different modalities may promote performance.

There are many ways to fusion images from different modalities. Image-level fusion is also widely called early fusion, and feature-level fusion is called late fusion. The result-level fusion is usually regarded as a post-process; in common sense, it is not better than the former two fusion methods. In our experiments, we found that image-level fusion works worse than all other methods and is even worse than a single modality. This result concludes that pre-trained weights are essential for deep networks and the input modalities are not precisely aligned. The seven patterns in our model are roughly the same, but if we look closely we can see that some patterns don’t match up exactly. This is because the convolutional layers are computed in small local areas, so the feature maps extracted in the image-fusion method do not have exact physical information.

Summing up or keeping the max value of the features both gave good results. But the components extracted from different networks represent different latent spaces. Therefore, we should assume that all parts of other modalities were in the same latent areas before every element of the feature vectors summed up or done max pooling. Concatenating all feature vectors seems more interpretable because different spaces can be connected into one higher dimensional feature space. However, the attached performance was not significantly good, and this method consumes much memory of GPU for the very high dimensional features.

Our proposed disentangling method targets this problem. It disentangles the features into two spaces, a modality-specific space, and a content space. Content space is shared between different modalities because we add constraint functions. Modality-specific space features are also trained with a constraint to keep them from each other to form a concise feature space. This fusion method combines the benefit of concatenating method and summing up the process, and the results prove that it performs better.

### Benefits from deep learning method

Deep learning methods have many advantages compared to traditional methods. Firstly, though they need much time for training, the inference process is super-fast. It takes only 0.2 seconds to process a whole series of 7 seven images, while the traditional takes about 120 seconds much time. This powerful processing speed makes it possible to quickly check many patients in a short time and even possible to give a real-time suggestion when taking MRIs. Furthermore, after taking MRIs, the proposed method can provide a diagnosis suggestion to radiologists or the MRI operators for whether the patients are probably suffering from highly cancerous mucinous cystic neoplasms.

Secondly, the deep learning method can be fully automatic in inference. Traditional radiomics needs to circle out the cysts, and it needs radiologists to find the center slices first. While the deep learning method requires only a position, the position can also be automatically detected using detection methods such as Faster RCNN17. Usually, radiologists have no time to help the machine segment out the area of the cyst for radiomics analysis.

Thirdly, the accuracy of the deep learning method is higher in our dataset. Conservatively speaking, deep learning methods work comparable to human experts and radiomics methods. Especially when not many experienced experts are available, the deep learning method can be regarded as a reliable assistant. PCN is not a frequent meet disease for doctors to accumulate experience and the classification between MCNs and SCNs is not easy for inexperienced doctors. In our experiments, the two experienced experts have PCN diagnosis experience for more than eight years. The proposed method can work comparable to them, which means for most doctors, advice from the proposed method is pretty helpful.

### Limitations

Since SCN and MCN are rare, the cohort we used was negligible. The images were obtained from different MRI facilities, and the acquisition time of the sequence may be slightly different owing to the various MRI operating staff. Besides, other types of disease which are also easier to misdiagnose with SCNs or MCNs can be added to experiments. Studies with more patients, more pancreatic neoplasm types, consistent scanning protocol, and much more complex networks and algorithms should be conducted in the future.

## Conclusion

This study effectively diagnoses SCN and MCN using a multi-modal hybrid deep learning network based on artificial intelligence algorithms. By utilizing a fusion structure that combines the features of seven modalities of MRI images, the research team demonstrated the performance of each modality through multi-modal MRI experiments on 69 patients. The proposed method achieved a high accuracy of 75% and an AUC value of 83% with optimized settings. This study indicates that utilizing modern artificial intelligence algorithms can enable physicians to achieve high performance in diagnosing complex SCN and MCN cases.

## Data availability statement

The relevant images and clinical data from this study are not available because they contain private patient information. However, such data can be obtained through agency approval and signed data use agreements and/or signed material transfer agreements. Requests to access the datasets should be directed to zhanggong301@126.com.

## Ethics statement

The studies involving humans were approved by Chinese People’s Liberation Army General Hospital Medical Ethics Committee. The studies were conducted in accordance with the local legislation and institutional requirements. Written informed consent for participation was not required from the participants or the participants’ legal guardians/next of kin in accordance with the national legislation and institutional requirements.

## Author contributions

GZ: Conceptualization, Methodology, Investigation, Writing – Original Draft WC: Methodology, Software, Formal analysis; ZW: Methodology, Investigation. FW: Visualization; JF: Resources, Data Curation. RL: Writing – Review & Editing, Supervision.
